# Biallelic *NDUFA4* Deletion Causes Mitochondrial Complex IV Deficiency in a Patient with Leigh Syndrome

**DOI:** 10.3390/genes15040500

**Published:** 2024-04-17

**Authors:** Doriana Misceo, Petter Strømme, Fatemeh Bitarafan, Maninder Singh Chawla, Ying Sheng, Sandra Monica Bach de Courtade, Lars Eide, Eirik Frengen

**Affiliations:** 1 Department of Medical Genetics, Oslo University Hospital and University of Oslo, 0450 Oslo, Norway; fbitarafan@yahoo.com (F.B.); ying.sheng@medisin.uio.no (Y.S.); eirik.frengen@medisin.uio.no (E.F.); 2 Division of Pediatrics and Adolescent Medicine, Oslo University Hospital and Faculty of Medicine, University of Oslo, 0450 Oslo, Norway; petter.stromme@medisin.uio.no; 3 Department of Neuroradiology, Oslo University Hospital, 0450 Oslo, Norway; mancha@ous-hf.no; 4 Department of Biochemistry, Oslo University Hospital and University of Oslo, 0450 Oslo, Norway; sanbac@ous-hf.no (S.M.B.d.C.); lars.eide@medisin.uio.no (L.E.)

**Keywords:** Alu element, COX, Complex IV, Leigh syndrome, encephalopathy, NDUFA4/COXFA4, WGS, structural variant (SV)

## Abstract

Oxidative phosphorylation involves a complex multi-enzymatic mitochondrial machinery critical for proper functioning of the cell, and defects herein cause a wide range of diseases called “primary mitochondrial disorders” (PMDs). Mutations in about 400 nuclear and 37 mitochondrial genes have been documented to cause PMDs, which have an estimated birth prevalence of 1:5000. Here, we describe a 4-year-old female presenting from early childhood with psychomotor delay and white matter signal changes affecting several brain regions, including the brainstem, in addition to lactic and phytanic acidosis, compatible with Leigh syndrome, a genetically heterogeneous subgroup of PMDs. Whole genome sequencing of the family trio identified a homozygous 12.9 Kb deletion, entirely overlapping the *NDUFA4* gene. Sanger sequencing of the breakpoints revealed that the genomic rearrangement was likely triggered by Alu elements flanking the gene. *NDUFA4* encodes for a subunit of the respiratory chain Complex IV, whose activity was significantly reduced in the patient’s fibroblasts. In one family, dysfunction of NDUFA4 was previously documented as causing mitochondrial Complex IV deficiency nuclear type 21 (MC4DN21, OMIM 619065), a relatively mild form of Leigh syndrome. Our finding confirms the loss of NDUFA4 function as an ultra-rare cause of Complex IV defect, clinically presenting as Leigh syndrome.

## 1. Introduction

The mitochondrial respiratory electron transport chain (ETC) is essential to carry out the oxidative phosphorylation (OXPHOS) process, resulting in the generation of ATP, the major source of cell energy, and regulates a spectrum of physiological processes, such as cell death, the production of reactive oxygen species (ROS), inflammation responses, tumor growth, and thermogenesis. This system is therefore critical to cell metabolism, and germline pathogenic variants in genes coding for components that are required for the OXPHOS process underlie “primary mitochondrial disorders”, PMDs [1], which cause a spectrum of metabolic conditions. The minimum birth prevalence of PMDs is estimated to be 1 in 5000 [2]. These diseases vary in terms of age of onset, rapidity of progression, and organ system involvement. Biochemical changes, myopathy, and neurological anomalies are often co-existent [3]. The spectrum of phenotypes is wide and includes, for example: deafness, blindness, liver failure, kidney disease, peripheral neuropathy, hormone deficiency, anemia, diabetes mellitus, and skin and hair anomalies [1]. Childhood progressive encephalopathies, also those caused by mitochondrial defects, carry an increased mortality risk, particularly if the onset occurs early in life [4]. The complexity of the clinical manifestations poses a challenge in the recognition of PMDs on clinical grounds. Similarly, the genetic bases of PMDs are heterogeneous with about 400 nuclear and 37 mitochondrial DNA (nDNA and mtDNA) genes documented to cause these diseases [5]. The high number of genes involved reflects the molecular complexity of the mitochondrial ETC, which consists of five enzymatic complexes and two mobile electron carriers (ubiquinone and cytochrome c). Its function is to couple the oxidation of reducing equivalents entering the ETC to the establishment of a proton gradient across the inner mitochondrial membrane that in turn is used to phosphorylate ADP. The mitochondrial ETC Complex IV, or cytochrome *c* oxidase (COX), is the terminal enzyme of the ETC. COX is a heme-copper oxidase, composed by 14 subunits where three mtDNA encoded subunits (MT-CO1, MT-CO2, and MT-CO3) form the catalytic core and 11 nDNA encoded subunits (COX4, COX5A, COX5B, COX6A, COX6B, COX6C, COX7A, COX7B, COX7C, COX8, and NDUFA4/COXFA4) are important for COX stability and regulation [6]. NDUFA4 was initially described as a Complex I subunit but was later resolved to be a protein capable of associating both to Complex I and Complex IV, although its role seems to be critical to maximizing Complex IV enzymatic activity [6,7,8,9,10].

The main aim of this study was to document that mutation in *NDUFA4* mediates ETC deficiency in an unreported patient with mitochondrial Complex IV deficiency nuclear type 21 (MC4DN21, OMIM 619065). Whole Genome Sequencing (WGS) analysis unraveled complete loss of the *NDUFA4* gene, and we documented significantly reduced COX activity in primary fibroblasts from the patient; these findings explain the patient’s symptoms in line with Leigh syndrome.

## 2. Materials and Methods

Genetic studies: The sample DNA was extracted from the whole blood and the whole genome library preparation was performed with the Illumina TruSeq DNA PCR-free Library Prep Kit (Illumina, San Diego, CA, USA). WGS was then performed with the NovaSeq 6000 system (Illumina, San Diego, CA, USA). The sequence reads were aligned with the human reference genome (hg19), and the SNP, indel, and CNV were called by the Illumina Dragen DNA pipeline (v3.9.3). The median coverage of the sample was 62×, with 89.44% of the genome covered by more than 20 reads and 89.79% covered by more than 10 reads. The coverage per gene was calculated by the tool DepthOfCoverage in the Genome Analysis Toolkit (GATK, v3.8.1) [11]. For Sanger sequencing of breakpoints, we designed the following primers using Primer3Plus Fwd TGTGGACAGGCATTGCTTTA and Rev TCCACTAACTTCAATGTGCAAAA to amplify the deleted allele, and Fwd GGTAAGGAGCATGGCTGTG and Rev ACCATGGCTTCTTTTTCCAG. DNA sequencing of the PCR products was performed on ABI 3730 with the ABI PRISM BigDye Terminator v. 3.1 sequencing kit (Applied Biosystems, Thermo Scientific, Waltham, MA, USA). Sequencing chromatograms were analyzed using CodonCode Aligner software version 8.0.2 (CodonCode Corp., Centerville, MA, USA).

ETC analyses: Fibroblasts were grown in DMEM/high glucose supplemented with 10% FCS. One million subconfluent cells were pelleted, washed in SETH (250 mM Sucrose, 2 mM EDTA, 10 mM Tris, 5 × 10^4^ U/L heparin, pH 7.4), and stored at −80 °C until their analyses. Whole cell lysates were used for the enzymatic analyses. Activities of citrate synthase (CS), and the electron transport chain (ETC) activities of Complex I (NADH-quinone oxidoreductase; NQR), Complex II/succinate dehydrogenase (SDH), NADH-cytochrome C reductase (NCR), succinate-cytochrome C reductase (SCR), and cytochrome C oxidase (COX) were performed according to Wibom et al. [12], and adapted to robotized analysis on IndikoTM Clinical Chemistry Analyzer (Thermo Scientific, Waltham, MA, USA). Briefly, we used 1 million cells (of separate passages) and prepared freeze–thawed cell lysate as described [12]. The established procedure has a detection limit (based on signal-to-noise ratio) for each complex. In 2 out of 6 preparations, the amount of extracted mitochondrial complex was below the detection limit for NQR activity and was consequently excluded. All complexes were related to citrate synthase activity, which means that this exclusion does not influence the range of activities. NQR and SDH activities were determined as the rotenone- and malonate-sensitive oxidation of NADH and succinate, respectively. The results shown are presented relative to the average of the activities in fibroblasts from two healthy donors.

## 3. Results

### 3.1. Clinical Description

The patient was a female born to first degree Somali cousins. Her parents and her elder sister were healthy. Pregnancy was normal until 7 months when fetal growth reduction was suspected. Delivery was by caesarian section at 38 weeks; weight was 3.2 kg, length 47 cm, and head circumference 36 cm, while Apgar scores were 9 and 10 after 1 and 5 min, respectively. Initial motor development was within the normal range, but the mother was worried at an early stage because she thought the girl’s development seemed somewhat slow besides having a “noisy” respiration. She walked independently at 18 months, but the staff in the kindergarten noticed that she seemed easily tired and showed less energy compared to her peers. At 2 years of age, she was referred to hospital because of unsteadiness, frequent falls, and inward rotation of her feet.

When seen again at age 2 years and 9 months, both language and motor skills were delayed. She had signs of cerebellar ataxia with intention tremor in her hands, and difficulty walking down the stairs. Muscle tone was increased, and the legs turned into varus position, indicating dystonia. She also manifested pyramidal tract involvement with generalized hyperreflexia, ankle subclonus, and inverted plantar reflexes. 

Language skills assessed with the New Reynell Developmental Language Scales at 2 years and 10 months were at the level of 1.5–2 years. When seen again at regular follow-up consultations, her condition had stabilized, without further loss of psychomotor skills. At the latest check-up aged 3 years and 9 months, there might even have been slight improvement, although easily provoked tiredness, unsteadiness, and cerebellar ataxia (Videos S1 and S2) with intention and action tremor in her hands had persisted. Cardiologic examination had not shown any structural abnormalities, but the ventricular walls were measured at the higher end of the normal range. Chest X-ray showed normal lungs and a borderline large-sized heart. Throat filming performed due to frequently provoked coughing when drinking water did not reveal any abnormal swallowing. Head circumference, height, and weight had followed the third percentile. A body mass index of 13.8 kg/m^2^ indicated slight underweight and prompted an energy-rich diet.

**Figure 1:**
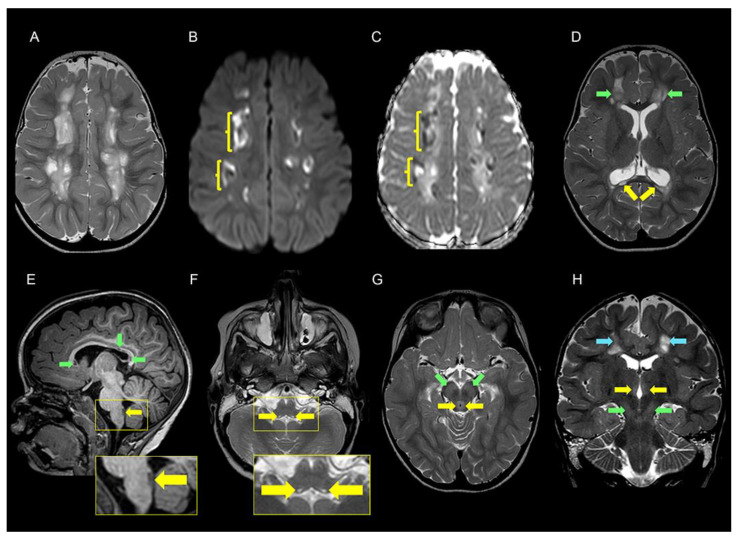
Cerebral MRI examination of the patient at the age of 2 years and 9 months. (**A**) T2 weighted axial image at the level of the centrum semiovale shows multiple lesions with varying degrees of high signal intensity typical of cavitary leukoencephalopathy. (**B**,**C**) Axial diffusion-weighted magnetic resonance imaging (DWI) (b1000 (**B**) and apparent diffusion coefficient (ADC) maps) (**C**) at the level of the centrum semiovale shows diffusion restriction along the margins (yellow parenthesis) of the cavitary leukoencephalopathy. (**D**) T2 weighted axial image at the level of corona radiata shows high signal of cavitary leukoencephalopathy in both frontal lobes (green arrows) and corpus callosum cysts (yellow arrows). (**E**) T1 weighted sagittal image shows thinning of the corpus callosum with low intensity foci compatible with cystic formations (green arrows). In the dorsal aspect of medulla oblongata, there is a 7 mm elongated low-signal lesion (yellow arrows). A magnification of this area is displayed in the rectangle below. (**F**) T2 weighted axial image at the level of the upper part of the medulla oblongata shows symmetric minute high signal changes (yellow arrows). An enlarged image of the area is shown in the rectangle below. The lesions correspond to the dorsal lesion displayed in the rectangle in E and are localized in close proximity to the dorsal motor nuclei of the vagus nerve. (**G**) T2 weighted axial image at the level of the mesencephalon shows high signal of periaqueductal gray matter (yellow arrows) and subtle increased signal in the substantia nigra on both sides (green arrows). (**H**) T2 weighted coronal image shows subtle high signal in the medial parts of both thalami (yellow arrows) and substantia nigra (green arrows) on both sides. There are also signal changes in the cerebral white matter at the level of centrum semiovale on both sides (blue arrows).

Neuroradiological examination. A brain MRI at 2 years and 10 months showed extensive widespread white matter pathology in both cerebral hemispheres. The signal changes differed in intensity giving the impression of numerous cysts or cavities in a “sea of leukoencephalopathy”. Corpus callosum was thin and contained cystic lesions. There were also signal changes in the mesencephalon, pons, and medulla oblongata (Figure 1). Magnetic resonance spectroscopy (MRS) was not performed. 

Laboratory tests. Spinal fluid lactate (measured once) was 3.6 mmol/L (reference 0.8–2.8 mmol/L). Plasma lactate values (measured three times) were 1.5, 4.2, and 2.1 mmol/L (reference 0.5–2.3 mmol/L). Creatin kinase (CK) values measured three times were 102, 145, and 224 U/L (reference 30–200 U/L). A slight elevation of CK could indicate some degree of muscular involvement. Plasma phytanic acid was mildly elevated to 19.3 µmol/L of unknown significance (reference 1.0–11.9 µmol/L). No other metabolic abnormalities were detected.

### 3.2. Genetic Findings

**Figure 2:**
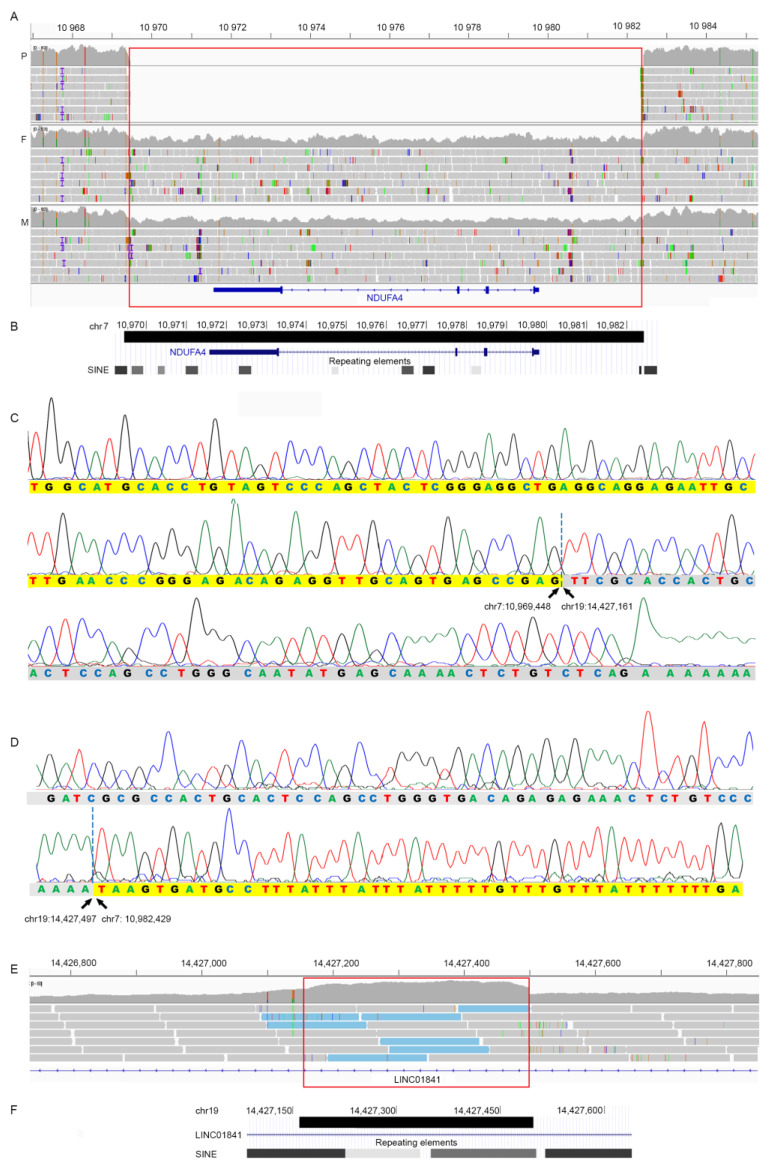
(**A**) WGS data of the patient (P), her father (F), and mother (M) showing the homozygous 12.9 Kb deletion (red box) on chromosome 7 overlapping the entire *NDUFA4* gene seq[GRCh37]del(7)(p21.3) NC_000007.13:g.10969473_10982428. The parents were heterozygous for the deletion (note the decreased read depth). Genomic positions are in Kb. (**B**) Modified screenshot from UCSC genome browser showing the deletion (black bar) chr7:10,969,448–10,982,429 and the presence of Alu elements at its borders. Genomic positions are in Kb. (**C**) Sanger sequencing of the PCR product amplifying the proximal breakpoint of the chr7 deletion. Bases highlighted in yellow identify sequence from chr7, bases in grey identify sequences from chr19, which start at chr19:14427161. (**D**) Sanger sequencing of the PCR product amplified at the distal breakpoint of the chr7 deletion, juxtaposed to chr19:14,427,497. In (**C**,**D**), bases highlighted in yellow and grey identify sequences from chr7 and chr19, respectively. (**E**) WGS data from the patient showing increased read depth in the region from chr19:14,427,161–14,427,497 indicating a duplication (red box). This sequence was found inserted at chr7:10,969,448. Note that the reads with a paired end mapping at chr7 are indicated in light blue. (**F**) Modified screenshot from UCSC genome browser showing the duplicated region (black bar) at chr19:14,427,161–14,427,497 and its overlaps with Alu elements.

Analysis of the WGS trio data identified the following deletion in heterozygosity in both parents and it was homozygous in the patient: seq[GRCh37]del(7)(p21.3) NC_000007.13:g.10969473_10982428 (Figure 2A). The 12.9 Kb deletion covers the entire *NDUFA4 (COXFA4)*, causing complete loss of the gene encoding NDUFA4 (Figure 2B). We PCR amplified and Sanger sequenced the breakpoint of the deletion in the patient (Supplementary Materials). This region was found to have sequence identity to three distinct genomic regions: 1. chr7:10969363–10969448, remapping the proximal breakpoint to chr7:10969448; 2. chr19:14427161–1442749, juxtaposed to chr7:10969448; and 3. chr7:10982429–10982473, confirming the distal breakpoint of the deletion at chr7:10982428 (Figure 2B–D). Therefore, the patient had a deletion of chr7:10969448–10982428, with 337 bp insertion from chr19:14427161–14427497. Retrospective analysis of the WGS data showed increased read depth for these 337 bp, indicating that there was a duplication of the 337 bp and an insertion at chr7:10969448 (Figure 2E). The 337 bp from chr19 consists almost exclusively of SINE elements: AluSq, AluJb, and AluJr (Figure 2F). Similarly, the proximal breakpoint at chr7:10969448 overlaps an AluSc, while the distal breakpoint maps 15 bp upstream of an AluSc8. All these repetitive elements share a high degree of sequence homology (Supplementary Materials). 

**Figure 3:**
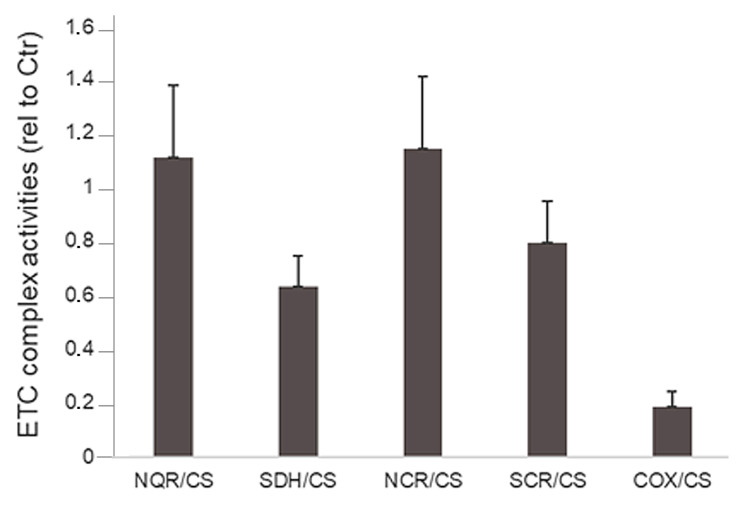
The ETC complex activities in fibroblasts from the patient presented relative to those in control fibroblasts. The activities were measured in whole cell lysates as described in the Materials and Method. Cells from 3 independent cultivations were used. COX/CS activity is significantly lower than control values (*p* = 0.005). For NQR/CS and SDH/CS: *n* = 2.

In line with the complete loss of *NDUFA4* in the patient, early onset neurological regression accompanied by cystic leukoencephalopathy and modest lactic acid elevation in CSF and plasma raised suspicion of mitochondrial dysfunction. Subsequent studies of the electron transport chain (ETC) in patient fibroblasts revealed significantly reduced cytochrome oxidase/citrate synthase (COX/CS) activity compared to controls (*p* = 0.005) (Figure 3).

NDUFA4 was previously documented to cause mitochondrial Complex IV deficiency nuclear type 21 (MC4DN21) with autosomal recessive inheritance in one family. The significantly reduced COX activity in fibroblasts from our patient allowed us to classify the homozygous deletion on chromosome 7 as pathogenic and causative of the clinical presentation of the patient.

## 4. Discussion

We describe a 4-year-old Somali female presenting with developmental delay, ataxia, dystonia, spasticity, extensive white matter signal changes, and metabolic abnormalities measured in CSF and plasma. The patient was homozygous for a 12.9 Kb deletion entirely overlapping *NDUFA4.* Sanger sequencing of the chr7 deletion breakpoints redefined the deletion’s proximal breakpoint and revealed that the deleted region was replaced by a 337 bp insertion from chr19:14427161–14427497. The 337 bases on chromosome 19 and the breakpoint’s regions on chr7 contain sequences of the Alu family (Figure 2). The Alu elements are an abundant family of sequence elements that has been amplified by retrotransposition during evolution to represent 10% of the human genome. These elements provide a major source of genome variability, contributing to both evolution and diseases. Alu elements, due to their high copy number and sequence homology, represent very dynamic and unstable genomic regions and may cause genetic diseases. The regions are prone to structural rearrangements through different mechanisms, for example, nonallelic homologous recombination (NAHR) or the Fork Stalling and Template Switching (FoSTeS) model [13]. The sequence homologies (Supplementary Materials) between the Alu elements at chr19:14427161–14427497 and those at the breakpoint’s regions on chr7 have likely triggered the deletion–insertion found in the present family. 

*NDUFA4* encodes for a protein important for COX stability and regulation. In line with this, significantly reduced COX activity was found in fibroblasts from the patient compared to controls, while the activity of Complex I was within the normal range, further supporting the notion that NDUFA4 is a Complex IV subunit [6,7,8,9,10]. However, it has to be noted that *NDUFA4* has two reported paralogues: *NDUFA4L2* [14] and C15orf48 [15], which replace NDUFA4 in a cell- and condition-specific manner and, potentially, could serve a role as a backup function in some cells in this patient. 

We did not find that the mouse knockout model for *Ndufa4* was characterized, but a Cre-Lox neuronal knockout was recently reported, where the authors found cellular abnormalities in the cortex, hippocampus, and cerebellum, and calcifications in the cerebellum [16]. These mice manifested impaired spatial learning capacity and exploratory activity [16].

Mitochondrial Complex IV deficiency, nuclear-type (MC4DN1, OMIM PS220110), or COX deficiency, is a genetically heterogeneous group of disorders with autosomal recessive inheritance caused by defects in more than 20 nDNA genes [17]. These genes encode for constituent subunits of COX and for proteins involved in the assembly of COX, for example, chaperones, transcriptional and translational activators, metal-binding proteins, and enzymes that complete the biosynthesis and incorporation of the heme group. In addition to the nDNA genes, mutations in the mitochondrial encoded genes *MT-CO1-3* also cause COX deficiency, and their clinical presentation and severity are largely dependent on the level of heteroplasmy [17]. COX deficiency manifests at an early age, often below 2 years, and can be lethal in childhood. However, mildly affected patients can have a later onset of symptoms and survive into adulthood, and intrafamilial variability has also been reported. The clinical phenotypes vary from isolated benign myopathy to infantile fatal form with multiorgan involvement [17]. The Leigh syndrome spectrum is a genetically heterogeneous PMD, caused by the dysfunction of one of 113 genes [18], including some related to *COX: MT-CO1*, *MT-CO2*, *MT-CO3*, *COX4I1*, *COX8A*, *COX10*, *COX15*, *LRPPRC*, *PET100*, *PET117*, *SCO2*, *SURF1*, *TACO1*. In addition, one homozygous loss of function variant in *NDUFA4* was reported to cause mitochondrial Complex IV deficiency nuclear type 21 (MC4DN21, OMIM 619065) in one family where the four affected family members had phenotypes not unlike our patient, as all had a clinical diagnosis of LS [8]. The patients described by Pitceathly et al. [8] were homozygous for *NDUFA4* c.42+4G>C, which caused aberrant splicing, and NDUFA4 was not detected in muscle tissue and cultured skin fibroblasts. In blood, but not in fibroblasts, low levels of the wildtype transcript were detected [8]. The low-level expression of the wildtype *NDUFA4* transcript was suggested to be responsible for relatively mild phenotypes with slow progression and survival into adulthood [8]. A clinical comparison of all the patients with pathogenic variants in *NDUFA4* is provided in Table 1. In the present patient, however, the entire gene was deleted, completely abolishing *NDUFA4* expression.

## 5. Conclusions

In summary, we present a second independent patient with MC4DN21, which is an ultra-rare genetic cause of LSS. The complete loss of NDUFA4 in our patient seemed to lead to more extensive leukoencephalopathy and brain stem lesions associated by neurological deficits typical of LSS and clinical presentation before the age of 2 years. The Alu repeat elements flanking the *NDUFA4* gene may provide sites of recurrent structural variation, which should be explored carefully in patients with LSS of unknown etiology. Identification of additional patients with NDUFA4 deficiency will further delineate the genotype–phenotype correlation in MC4DN21.

## Figures and Tables

**Table 1:** Phenotype in patients with biallelic loss of function variants in *NDUFA4*.

Patients	Patient 1	Four Patients Reported in the Literature [8]
Age at last examination; gender	4 y; female	2 females (II-3 32 y; II-6 26 y, died), 2 males (II-4 34 y; II-13 8 y, died)
Country of origin	Somalia	Pakistan
Perinatal history	Normal	Low birth weight (2/4), poor growth (2/4), resuscitation (1/4)
**Neurological functioning**		
Cognition	Language delay, learning difficulties	Language delay, learning difficulties (4/4)
Motor skills	Delayed; regression followed by slight improvement	Delayed (3/4); regression from age 4 y (1/4)
Pyramidal tract signs	Hyperreflexia, spasticity	Not reported
Extrapyramidal signs	Dystonic positioning of the feet	Dystonia (3/4)
Cerebellar signs	Ataxia, intention- and action tremor, broad-based gait	Ataxia (2/4), nystagmus (1/4)
**Lactate measurements**		
CSF	Elevated ^1^	Not reported
Plasma	Elevated ^2^	Congenital lactic acidosis (4/4)
**Cerebral MRI**		
Cerebral hemispheres	Leukoencephalopathy, widespread with multiple cavities ^3^	Scattered signal changes in deep white matter
Corpus callosum	Atrophy, multiple cysts ^4^	Not reported
Brain stem and mesencephalon	Signal changes in pons and medulla oblongata ^5^ and periaqueductal grey matter ^6^	Signal changes in pons and medulla oblongata
Basal ganglia	Signal changes in substantia nigra and thalami ^7^	Signal changes in medial thalami
Cerebellum	Normal	Signal changes in cerebellum and right middle cerebellar peduncle
**Diagnosis**	Leigh syndrome	Leigh syndrome (4/4)

**Legend**. ^1^ 1/1 measurement; ^2^ 1/3 measurements; ^3^ Figure 1A–D; ^4^ Figure 1D,E; ^5^ Figure 1E,F; ^6^ Figure 1G, ^7^ Figure 1H.

## Data Availability

The data will be available upon request. The distribution of sensitive data may be subject to restrictions.

## Supplementary Materials

The following supporting information can be downloaded at: https://www.mdpi.com/article/10.3390/genes15040500/s1, File S1; Video S1: Video of the patient at age 3 y 11 m shows action- and intention tremor; Video S2: The video shows impaired balance and dystonic positioning of the feet.
