# Supplementary material for: Biallelic NDUFA4 Deletion Causes Mitochondrial Complex IV Deficiency in a Patient with Leigh Syndrome
Source: Genes (Basel). 2024 Apr 17;15(4):500. doi: 10.3390/genes15040500 (PMC11050323; doi:10.3390/genes15040500)
Supplement: Supplementary file 1 [file genes-15-00500-s001.zip › Misceo NDUFA4 Supplemental 13032024.pdf]

Supplemental material

Biallelic *NDUFA4* deletion causes Mitochondrial Complex IV deficiency in a patient with Leigh syndrome

Sanger sequencing of the PCR product obtained with primers flanking the deletion in the patient

```
ATCATCCCAC TTCTTAAGAC TTACAGCTCT GGTGTAAAAG CCAGTCACCT 50
TGTTCATGTG TTGAAATCAA TGATATTTGC AATAAAAAGA TTATTTAATC 100
AGATTGTTAA CAGCTGAATA AGCTTTTACC AAATAGTCCT TTTCTTTCCA 150
AGTTTTATAT AATATGGCTT TTAGAATTTT CCCCgccagg catggtggct 200
cacgcctgta atcccagtac ttcaggaggt caaggcagga agatcacgac 250
gtgaagagac tgagaccatc ctggccaata tggtgaaaac tcgtctctac 300
caaaaataca aaaaattacc ccagtgtggt ggcatgcacc tgtagtccca 350
gctactcggg aggctgaggc aggagaattg cttgaacccg ggagacagag 400
gttgcagtga gccgagttcg caccactgca ctccagcctg ggcaatatga 450
gcaaaactct gtctcagaaa aaaaaaaaag aattgcttga actcaggaga 500
tagaggttgc agtgagccaa gattgcacca ctgcactcca actgggtgac 550
agagtgagac tccatctctg aaaacaaaat aaaataaaaa TAACAAAGAA 600
AACCAgctgg gcatgttggc ataaccgtag tcccacctac ttgggaggct 650
aaagcgggag gattgtttaa actcaggagt tgcaggctac aatgaactaa 700
gatcgcgcca ctgcactcca gcctgggtga cagagagaaa ctctgtccca 750
aaaTAAGTGA TGCCTTTAtt tatttatttt tgtttgttta ttttttgag 800
atggagtctc gctctgtggc ccaacctgga gtgtggtggc acgatctcgg 850
ctcactgcaa cctctgcctc ccaggttcaa gcgattctcc tgcctcagcc 900
tccccaatag ctgggactac aggcacatgc caccacaccc ggttaatttt 950
ttgtattttt agtagagatg ggtttcacc gtgttagtca ggatggtctc 1000
gatctctgga cctcgtgatc tgcccacctc ggcctcccaa agtgctggga 1050
ttacaggtgt gagccaccgc acctggccTA AGTGATGCCT TTAGAGATGT 1100
TAACAAAGCA AGGTTACAAA TATAAATTTC ATTAAATATT AACCAAAATG 1150
TCAAATTGTG AAAGTTTAAA TTTTGCACAT TGAAGTTAGT GGA
```

Color legend.

Black DNA mapping at chr7:10969033-10969448 with 100% identity.

Blue DNA mapping at chr19:14427161-14427497, 337 bases, with 100% identity.

Green DNA mapping at chr7:10982429-10982868 with 100% identity.

The 337 bases have also 82.1% identity to chr7:101018817-101019244, spanning 428 bases (https://genome.ucsc.edu/, BLAT Tool).

Small letters indicate repetitive elements.

Alignment between chr19:14427161-14427497 and AluSc8 at chr7:10982444-10982753

Identities	Gaps	Strand
91/116(78%)	2/116(1%)	Plus/Minus

```
Chr19:14427223 aGAATTGCTTGAACTCAGGAGATAGAGGTTGCAGTGAGCCAAGATTGCACCACTGCACTC Chr19:14427282
               ||||| ||||||||   ||||  ||||||||||||||||| |||| |  ||||  |||||
Chr7:10982563  AGAATCGCTTGAACCTGGGAGGCAGAGGTTGCAGTGAGCCGAGATCGTGCCACCACACTC Chr7:10982503

Chr19:14427283 CA-ACTGGGTGACAGAGTGAGACTCCATCTCTGaaaacaaaataaaataaaaataa  Chr19:14427337
               ||   ||||  |||||| |||||||||||||  |||| | ||  ||| ||||||||
Chr7:10982503  CAGGTTGGGCCACAGAGCGAGACTCCATCTC-AAAAAAATAAACAAACAAAAATAA  Chr7:10982449
```

Alignment between chr19:14427161-14427497 and AluSc at chr7:10969217-10969522

Range 1: 239 to 296

Identities	Gaps	Strand
52/60(87%)	2/60(3%)	Plus/Plus

```
Chr19:14427167 CCACTGCACTCCAGCCTGGGCAATATGAGCAAAACTCTGTCTCAGaaaaaaaaaaaaGAA  Chr19:14427226
               ||||||||||||||||| ||||| | |||||| ||||  ||||| |||||||||||| ||
Chr7:10969455  CCACTGCACTCCAGCCT-GGCAACA-GAGCAAGACTCCATCTCAAAAAAAAAAAAAAAAA  Chr7: 10969513
```

Range 2: 190 to 304

Identities	Gaps	Strand
95/115(83%)	0/115(0%)	Plus/Plus

```
Chr19:14427223 aGAATTGCTTGAACTCAGGAGATAGAGGTTGCAGTGAGCCAAGATTGCACCACTGCACTC  Chr19:14427282
               |||||||||||||| | ||||| ||||||||||||||||| |||| |  |||||||||||
Chr7:10969406  AGAATTGCTTGAACCCGGGAGACAGAGGTTGCAGTGAGCCGAGATGGTGCCACTGCACTC  Chr7:10969465

Chr19:14427283 CAACTGGGTGACAGAGTGAGACTCCATCTCTGaaaacaaaataaaataaaaataa  Chr19:14427337
               || |  ||  ||||||  ||||||||||||  |||| |||| |||| ||||| ||
Chr7:10969455  CAGCCTGGCAACAGAGCAAGACTCCATCTCAAAAAAAAAAAAAAAAAAAAAAAAA  Chr7:10969513
```

Range 3: 143 to 283

Identities	Gaps	Strand
107/142(75%)	3/142(2%)	Plus/Plus

```
Chr19:14427358 TGTTGGCATA-ACC-GTAGTCCCACCTACTTGGGAGGCTAAAGCGGGAGGATTGTTTAAA  Chr19:14427415
               || ||||||  ||| ||||||||| ||||| |||||||| | || |||| |||| || ||
Chr7:10969359  TGGTGGCATGCACCTGTAGTCCCAGCTACTCGGGAGGCTGAGGCAGGAGAATTGCTTGAA  Chr7:10969408

Chr19:14427416 CTCAGGAGTTGCAGGCTACAATGAACTAAGATCGCGCCACTGCACTCCAGCCTGGGTGAC  Chr19:14427475
               | | ||||    ||| | || ||| |  |||| | |||||||||||||||||| ||  ||
Chr7: 10969409 CCCGGGAGACAGAGGTTGCAGTGAGCCGAGATGGTGCCACTGCACTCCAGCCT-GGCAAC  Chr7:10969458

Chr19:14427476 AGAGAGAAACTCTGTCCCAAAA  Chr19:14427497
               ||||  | ||||  || |||||
Chr7: 10969459 AGAGCAAGACTCCATCTCAAAA  Chr7:10969499
```
